# Proceedings of the BC Summit on Navigation for Children and Youth with Neurodevelopmental Differences, Disabilities, and Special Needs

**DOI:** 10.1186/s12919-022-00232-z

**Published:** 2022-05-17

**Authors:** Emily Gardiner, Vivian Wong, Anton R. Miller

**Affiliations:** 1 BC Children’s Hospital Research Institute, 950 West 28th Avenue, Vancouver, BC V5Z 4H4 Canada; 2 Division of Developmental Pediatrics, Department of Pediatrics, University of British Columbia, 4480 Oak Street, Vancouver, BC V6H 3V4 Canada; 3 Sunny Hill Health Centre at BC Children’s Hospital, 4500 Oak Street, Vancouver, BC V6H 3N1 Canada

**Keywords:** Neurodisability, Patient Navigation, Family Support, Summit, Participatory Action Research, Community Engagement

## Abstract

Patient navigation (PN) represents a branch of service delivery traditionally aimed at coordinating disjointed care services for patients with particular health conditions (e.g., cancer, HIV, diabetes). Over time, this approach has been extended to various social and health contexts, including most recently to children with neurodisability and their families. In this context, PN involves the provision of information, advice, education, and emotional support, coordination of services both within and across sectors, and the work is guided by person- and family-centred principles of practice. This manuscript documents the proceedings of the *BC Summit on Navigation for Children and Youth with Neurodevelopmental Differences, Disabilities, and Special Needs*, which took place on January 18 and 19, 2021 and was developed in collaboration with a Provincial Advisory Group. Our Summit brought together over 120 individuals, including researchers, government personnel, service providers, educators, healthcare workers, and family advocates. As part of the event, attendees learned from families with lived experience navigating the British Columbian (BC) service system, from BC Children’s Hospital Research Institute investigators, and from exemplar providers who deliver navigation services in various contexts (e.g., locally, regionally, and provincially). Attendees also participated in various engagement opportunities, and collaboratively identified directions for developing a future community of navigation and related services in BC.

## Patient navigation and children with neurodisability

Patient navigation (PN) is a branch of service delivery broadly focused on: (i) reducing barriers to access; (ii) connecting individuals and/or families to the services they need and desire; (iii) integrating varied services in such a way that they are coordinated and accessible, as opposed to siloed and disconnected; and (iv) providing information and emotional support to empower patients and families [1, 2]. Although PN originated two decades ago from Dr. Harold Freeman’s work with low-income breast cancer patients living in the Harlem area of New York City [3], the core concepts have been successfully applied across a wide range populations and health care contexts [4, 5].

Recently, there has been increased attention in extending PN programs to pediatric populations, and specifically to children with neurodisability (ND), which has been defined as:

a group of congenital or acquired long-term conditions that are attributed to impairment of the brain and/or neuromuscular system and create functional limitations. A specific diagnosis may not be identified. Conditions may vary over time, occur alone or in combination, and include a broad range of severity and complexity. The impact may include difficulties with movement, cognition, hearing and vision, communication, emotion, and behaviour ([6], p. 3–4).

The nature of childhood ND, in which children demonstrate functional difficulties over time and across developmental domains, means that a diverse array of supports must be accessed. Although these supports originate from distinct service sectors (e.g., health, social, education, employment, financial), there is often no accompanying individual or agency available to assist caregivers with the attendant coordination requirements. Indeed, caregivers report the extraordinary amount of time and energy spent to coordinate their child’s services, difficulty in obtaining information, and confusion around how best to ‘navigate’ amongst programs’ diverse access and eligibility criteria as burdensome and isolating [7].

Recent research highlights the benefit that PN programs can have for individuals with ND and their families in terms of facilitating improved service access, uptake, and satisfaction, as well as perceived benefits to quality of life [8–14]. Within the particular health context of childhood ND, PN tends to be a time-limited service, in which professional, lay, or peer navigators come alongside families in order to identify and address the barriers preventing them from successfully obtaining needed services and supports. Navigators provide information, advice, education, and emotional support, and coordinate resources within and across services, agencies, and systems [15].

The application of PN programs within Canada is relatively new, as applied both broadly [16] and specifically to childhood ND [15]. For example, in Luke et al.’s [17] environmental scan of pediatric PN programs across Canada, none specifically served children with ND, though six of the 23 identified programs had target populations that would encompass this group, indicating they served “all conditions”. In addition, there are emerging research-based Canadian initiatives that show great promise, such as NaviCare/SoinsNavi in New Brunswick, Canada, which provided navigation support to both families of children and youth with complex care needs and their care providers [8, 13].

## Integrated Navigational Support Program

Another such innovative Canadian initiative is the Integrated Navigational Support Program, which commenced in 2017. This participatory and community-based program is focused on both research and capacity building, and is aimed at understanding and addressing barriers preventing families of children with ND from accessing needed services. Across three Western Canadian sites –Alberta, Yukon, and British Columbia (BC) – researchers have partnered with community-based service providers, family advocates, government representatives, and ND advocacy organizations to identify region-specific barriers and establish project priorities and activities. In Alberta, this work has focused on partnering with stakeholders to produce training modules for navigators, and a pilot peer-to-peer mentoring program. In Yukon, this initiative has taken the form of funding a navigator position in a remote community (see [18] for a more detailed description of the overall project and site-specific initiatives).

In BC, the team has focused on provincial efforts to raise the profile of navigation and its importance to the ND community (see www.bcchr.ca/navigationproject). Our broad goal was to partner with relevant stakeholders to increase connectedness, collaboration, and information sharing amongst PN-related organizations serving children with ND and their families across the province. Specifically, in partnership with a Provincial Advisory Group (PAG) comprised of researchers, service providers, government personnel, and parent advocates (see Table 1), the project directed focus toward three main, actionable barriers, identified in a Problem Identification and Prioritization Process undertaken in 2018 by the PAG and BC researchers:

1. The role and scope of navigators and navigational work is not well defined in BC;
2. There is a lack of connectedness across agencies, including understanding of the roles and responsibilities of each, and how they intersect and potentially overlap; and
3. There is a lack of integration of service providers with navigator roles and functions, and this varies across communities and subpopulations of children*.*

**Table 1:** Provincial Advisory Group organizations represented and organizational aims

Organization	Aims
Aboriginal Supported Child Development (ASCD)	Designed to meet the needs of First Nation, Métis, and Inuit children who require additional support to be included in childcare settings, and their families. ASCD programs are developed with cultural values, beliefs, and traditions in mind.
Asante Centre	Offers assessment, diagnostic, and family support services for individuals and families who are, or are suspected of, living with fetal alcohol spectrum disorder, autism spectrum disorder, or other complex developmental needs.
Autism Information Services British Columbia (AIS BC)	Assists families of children with autism spectrum disorder, professionals, community partners, and government colleagues by providing information, resources, and training opportunities. AIS BC also promotes collaboration and coordination between provincial autism-related service organizations and community support providers, and manages and administers the Registry of Autism Service Providers (RASP).
British Columbia Association for Child Development and Intervention	Association representing non-profit agencies delivering services to children and youth with support needs and their families (e.g., community child development centres) across the province.
BC Centre for Ability	Provides early intervention, rehabilitation, child development, mental health, and employment services for children, youth and adults with disabilities across British Columbia.
BC Children’s Hospital Research Institute	Research institute affiliated with the BC Children’s Hospital conducting pediatric research with the goal of helping children reach their full potential.
Community Living British Columbia	A crown corporation that funds supports and services to adults with developmental disabilities, as well as individuals who have a diagnosis of autism spectrum disorder or fetal alcohol spectrum disorder and who also have significant difficulty doing things on their own.
Family Advocates	Parents of children with neurodisabilities, who also work with the Family Support Institute of BC.
Family Support Institute of BC (FSI)	Provincial not for profit society committed to supporting family members who have a family member with a disability. FSI provides peer support, workshops and training, parent networking opportunities, and information sharing and referral support.
Ministry of Children and Family Development	This provincial government ministry supports the well-being of children, youth and families in British Columbia by providing services that are accessible, inclusive, and culturally respectful.
Services to Adults with Developmental Disabilities	Offers navigator services for youth with developmental disabilities transitioning out of pediatric services, and their families. Navigators coordinate transition planning and access to supports and services through the transition period of 16–24 years old.
Sunny Hill Health Centre at BC Children’s Hospital	Provides specialized developmental assessments and rehabilitation services to BC children and youth and their families.

## BC Summit on Navigation for Children and Youth with Neurodevelopmental Differences, Disabilities, and Special Needs

Over the course of the project, engagements with stakeholders have confirmed the sentiment that those organizations providing navigation services in BC for families of children with ND are usually not well connected, and often unaware of each other. This gap results in navigation providers working in relative isolation, and experiencing uncertainty with knowing where to best direct families for specific support services. In order to address this gap (i.e., Barrier 2), the PAG planned and hosted a provincial Summit – the *BC Summit on Navigation for Children and Youth with Neurodevelopmental Differences, Disabilities, and Special Needs* (hereby referred to as the ‘Navigation Summit’). The goals of this event were to:

1. Deepen understandings of the importance of “navigation” and what “navigation” is;
2. Develop coalitions (including of families and stakeholders) and opportunities for interagency collaboration – possibly through a Community of Practice; and
3. Understand priorities for the future.

Over the mornings of January 18–19, 2021 and led by an event facilitator with content expertise, over 120 Summit attendees learned from families with lived experience navigating the BC service system, from BC Children’s Hospital Research Institute investigators, and from exemplar providers who deliver navigation services in various contexts (e.g., locally, regionally, and provincially, as well as condition-specific support). Due to the Covid-19 pandemic and associated provincial restrictions on large gatherings, the Summit event was held virtually via Zoom. American Sign Language interpretation was provided throughout all sessions and breakout rooms for participants who had indicated the need based on their being deaf of hard of hearing.

### Navigation Summit attendees

**Fig. 1:**
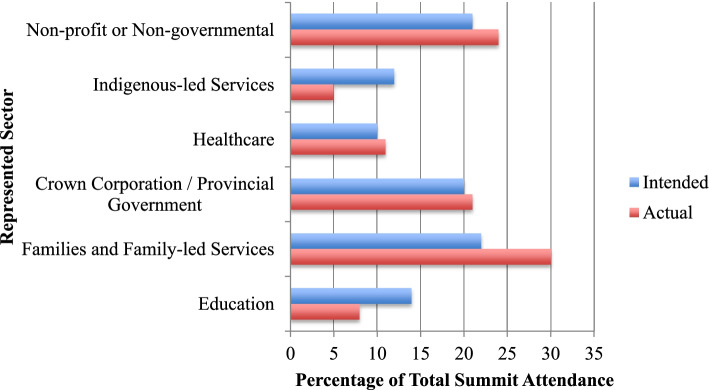
Intended and Actual Representation of Summit Attendees Across Sectors Relevant to Navigation

In planning the Navigation Summit, PAG members felt that the following three aspects were critical: that attendees be representative of service sectors relevant to children with ND; ensuring that no one group be overrepresented; and that family members had an equitable voice. The planning committee, which was comprised of the PAG and event facilitator, identified the relevant sectors as education, family-led services, healthcare, indigenous-led services, non-profit or non-governmental organizations, those providing navigation through a crown corporation or provincial government program within the Ministry of Children and Family Development, and family advocates. Based on personal and professional contacts, the planning committee collaboratively identified the relevant organizations and services within each highlighted sector, and ‘save the date’ emails containing preliminary event information were sent in October 2020. These emails gave recipients the opportunity to complete an online Expression of Interest form, within which respondents provided their name, contact information, and organizational affiliation. Based on these initial responses, the PAG assigned ‘seats’ to each of the identified sectors to ensure that the Navigation Summit would have varied and representative participation. Following this, formal invitations specifying the number of spots allocated to each organization and service provider were sent. In order to ensure families were represented at the event, the planning committee followed the Guidelines for Patient Partner Compensation indicated by CHILD-BRIGHT [19], and offered each attending family member an honorarium of $150, as well as to reimburse any incurred childcare expenses. The breakdown across sectors based on intended and actual final attendance is represented in Fig. 1, with further details about the priority populations and regions served by represented organizations presented in Table 2.

**Table 2:** Summit attendees’ served populations and provincial regions

	*n*
Priority Populations Served	
Rural and remote communities	68
Indigenous communities – First Nations, Inuit, or Metis families	77
Low-income or socioeconomically vulnerable	77
Newcomers to British Columbia and Canada	57
Provincial Regions Served	
Interior Health Authority	22
Vancouver Coastal Authority	19
Fraser Health Authority	20
Vancouver Island Health Authority	24
Northern Health Authority	18
Province-wide	50

Some participants endorsed multiple categories

### Navigation Summit content proceedings

#### Day 1 of the Navigation Summit

The Navigation Summit opened with an introduction and welcome from Dr. Anton Miller, Developmental Pediatrician and Clinician-Scientist with the Department of Pediatrics, University of British Columbia. Dr. Miller provided an overview of the Integrated Navigational Support Program, with specific attention to the work that had taken place in BC to date. He described the goals of the Navigation Summit and the content of each day.

##### Session 1. Learning from families

The first session on Day 1 began with family speakers sharing their stories and experiences of navigating amongst BC service systems in support of their children with ND. The first, Ashley Seltenrich, shared her experience as an Indigenous mother of a preschool-aged son with ASD living in a rural community. The second speaker, Brenda Lenahan, is a single mother of a school-aged child with a rare genetic condition, who also lives in a rural community. Brenda shared her perspective as a parent, but also as an advocate, as she founded the organization BC Parents of Complex Kids Society in 2017, which seeks to facilitate parent networking and advocacy for equitable support access across conditions. Finally, Si Stainton shared her perspective both as a mother of a young adult with a developmental disability and as a Family Services Manager with the Delta Community Living Society. Parent speakers shared a number of key insights, including the importance of professionals reaching out to families in order to reduce the family’s burden to initiate the connection to support, and about the range of supports that can benefit families, including tangible resources, such as funding and specialized equipment.

Following the Learning from Families session, Navigation Summit attendees were asked, “What’s the wisdom in these families’ stories we should carry forward?” Through Mentimeter (https://www.mentimeter.com), an interactive presentation tool that allows live polling from event participants, attendees provided anonymous comments that were shared in real time. One hundred and forty eight unique comments were provided in response to this question, and responses fell across three themes. First, attendees commented on the need to simplify the systems within which families navigate. There was also an expressed need for professionals to use plain language, and to be knowledgeable and up-to-date with their awareness of available services to which they can direct families. The second group of comments centered around the need for navigators to establish trust-based relationships with families that are sensitive to diverse cultural backgrounds, and for professionals to attend to their unique needs. Finally, the value of peer-to-peer connections was emphasized. Families have unique knowledge and perspectives to share, and this was perceived as a very meaningful form of support.

Attendees were then placed in small groups of 4–5 participants across 21 breakout rooms, within which they were asked what would help improve families’ experiences in navigating service and support systems. Using Miro (https://www.miro.com), an online collaborative whiteboard platform, individuals discussed and posted responses. Within these discussions, attendees emphasized the need for improved communication amongst professionals, and collaboration across government ministries and agencies, in order to reduce the need for families to coordinate disjointed services. They also expressed a desire for the establishment of networks and connections amongst service providers that would strengthen communication, and professional development opportunities to improve knowledge. Finally, attendees communicated a desire that the knowledge navigators share with families be both accurate and transparent, such that families are better informed as to what services and supports are available to them rather than having to seek this information themselves. They also emphasized the importance of peer support, as connections to other families reduce feelings of isolation.

##### Session 2. Learning from the research

**Fig. 2:**
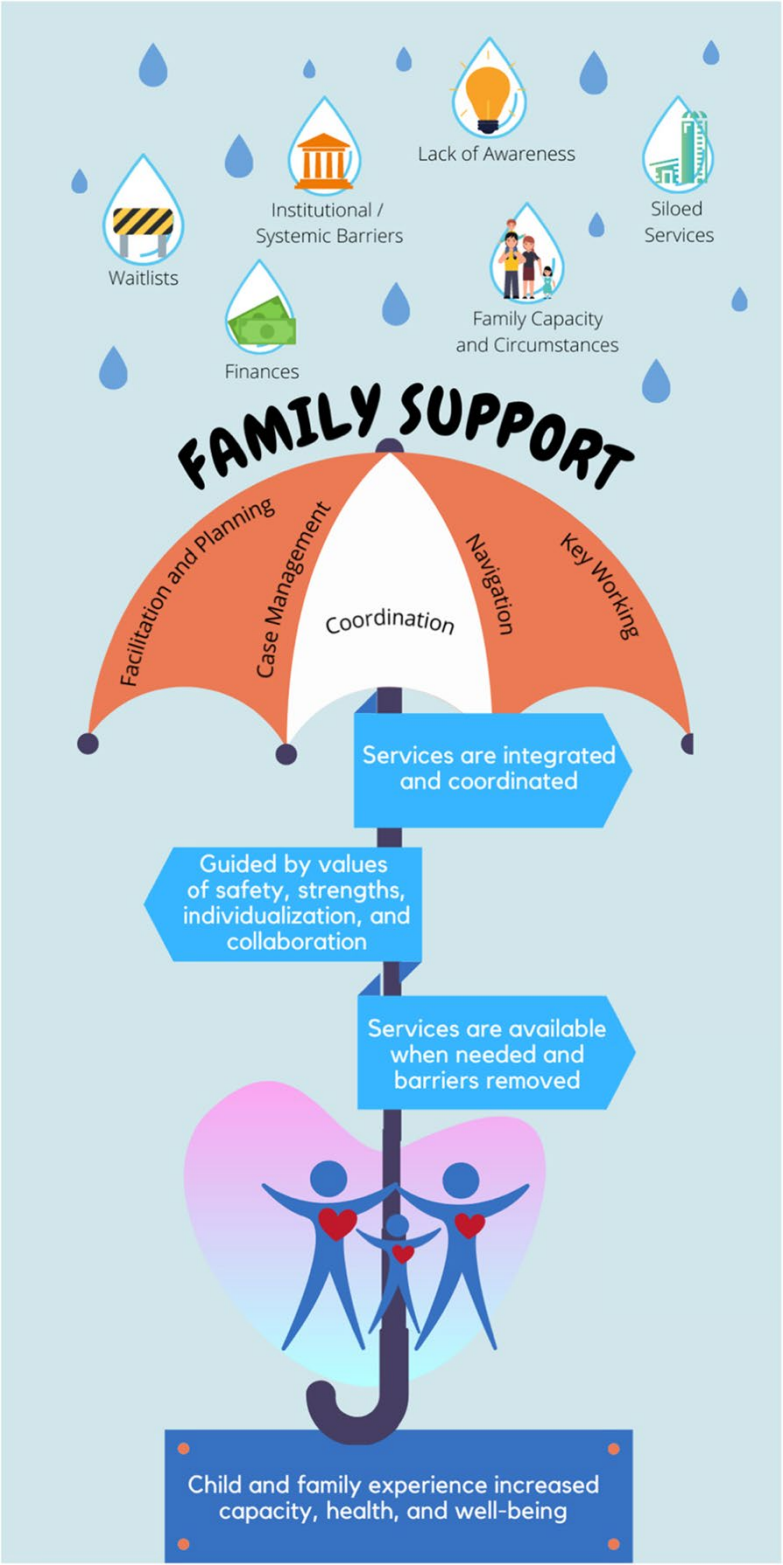
Summative Visual Integrating Findings from Navigation Research Shared with Summit Attendees

Prior to the Navigation Summit, attendees were sent a video featuring Dr. Emily Gardiner, Postdoctoral Research Fellow with the BC Children’s Hospital Research Institute and University of British Columbia Department of Pediatrics. Dr. Gardiner reviewed research findings from studies the BC research team had conducted examining terminology and role descriptions related to navigation for children with ND and their families (further details about this research and the findings can be found in Gardiner et al. [15] and Gardiner et al. (Gardiner, Wong, Miller: Navigators’ perceptions of their work in childhood neurodisability: What they do and what they call it, forthcoming). Event registrants also received a handout that included a summative visual integrating these study findings (Fig. 2). This figure illustrates the range of barriers families confront as they navigate amongst ND-related supports, the varied terms that are used to refer to these services, the principles and activities underpinning the work, as well as the ultimate goals. This Summit session consisted of 21 breakout rooms using Miro, within which attendees were asked “How does this visual and description of family support resonate with your experience and practice?” and “How can we go about devising an inclusive terminology that all stakeholders feel gets to the essence of this kind of family support work, and are comfortable to use and support?” In response to the visual, attendees suggested additional barriers that could be included, such as family legal challenges and histories of trauma. They also suggested additional values be included, such as being family-centred, trauma-informed, and strengths-based. With respect to developing an inclusive terminology, Summit attendees suggested that whatever term is used, it needs to be driven by parents and families, and be one that they connect and relate to. They also reflected that one term may not work for every family, but that adhering to plain language would be helpful.

#### Day 2 of the Navigation Summit

##### Session 1. Learning from providers

The first session on Day 2 consisted of presentations from three BC professionals who provide navigation supports in various capacities. These professionals spoke about their agency mandates, scope of their role, and the services and supports they offer to families. The first presentation was from Carly Owen, Autism Support and Resource Specialist with Autism Information Services BC (AIS BC), a provincial government program under the Ministry of Children and Family Development that offers information on best practice treatments and supports pertaining to ASD and related disorders, as well as training to families, service providers and professionals.

The second speaker was Angela Clancy, Executive Director of the Family Support Institute of BC (FSI), a provincial non-profit society that offers volunteer-based peer-to-peer support for family members of individuals with disabilities across the province, regardless of age or diagnosis. She described FSI’s organizational structure, her history with FSI, and their vision for connecting and valuing the family, and to support building communities for families. FSI is guided by a strengths-based perspective that recognizes the family’s expertise, and strives to empower families. FSI also offers workshops and training, all of which are written and delivered by families, parent networking opportunities, and information sharing and referral support.

Finally, Kimberlee Howland, Resource Family Navigator with the Nanaimo Child Development Centre spoke, describing the therapy services and programs offered at her centre, and her specific role. As a Resource Family Navigator, Kimberlee assists families navigating complex health and service systems (e.g., education, health, mental health, funding) by connecting them with resources, facilitating opportunities for parent networking, and for educational enhancement. She also shared her perceptions about what makes an effective navigator, including knowing your community and what is available, being connected to community and provincial organizations, being relationship-based, using positive and supportive communication, having healthy boundaries, maintaining an up-to-date database of both local and provincial services, and having access to team support for when particularly complex family situations arise.

##### Session 2. Creating strategies together for building a community of navigation and related services and supports in BC

The remainder of Day 2 was devoted to attendees considering what could be done to improve navigation supports and related activities for families, and to build a community of navigation. Using Miro, the event facilitator implemented a strategy whereby attendees themselves proposed room topics, and hence ‘co-designed’ this part of the Summit. In total, 19 topics were put forth (see Table 3 for the list of topics) and each topic had a dedicated breakout room created. Attendees then self-organized into the rooms that were of interest to them, and were able to move between topics as desired. Overall, a number of strong themes emerged within these self-organized conversations. First, attendees described wanting an up-to-date inventory of available services that would be easily accessible (e.g., a web-based portal), and applicable to families of children in various stages of development, from the early years through to adulthood. Navigators also strongly expressed that they wanted a way to become and remain connected to one another, such as through a community of practice or community-based working groups, noting that nothing like this exists in this area of service provision. These networking opportunities would give them a venue through which to ask questions, brainstorm solutions, share resources, and to develop best practice guidelines for their profession. They also emphasized the need for further or enhanced education, such as on cultural safety for serving Indigenous families, as well as ways for families to be better informed about what supports could be offered through case management, and to train them on advocacy. Attendees highlighted the strong need for the family voice to be represented, and for families to be able to connect with one another through peer support initiatives. Finally, there was an overwhelming desire for a simplified system of services and supports for children, young people and families affected by ND (e.g., “one stop shop”). These discussions referenced a need for flexibility in how families access support (e.g., not tied to specific diagnoses), collaboration across ministries, services, and systems, as well as transparency from government.

**Table 3:** Topics proposed by Summit attendees during the ‘*Creating strategies together for building a community of navigation and related services and supports in BC’* session

• It seems that there might be navigation services happening in a variety of forms out there already. Should we perhaps do an inventory of what’s out there to create some type of map of community based an provincial navigation supports?	
• How do we support Indigenous families in a culturally safe way?	
• Grants, funds and sustainability.	
• Having service providers meet by region to better serve families.	
• A community of practice for navigators -what would that look like and how should it be built?	
• More rural inclusion support/funding (Northern/West, Interior, Cariboo, Chilcotin). How can we have a stronger link to the north and rural communities?	
• How do we get funding support to support the vision of more advocacy / navigators for families?	
• Does anyone have news about how far the recommendation from the Select Standing Committee has gone for family navigators?	
• How do we identify those who navigate, but don’t identify as navigators?	
• How do we make fragmented systems more streamlined and collaborative? (i.e.: NSS AHP, CYSN, Therapy services, etc.)?	
• Establish a framework for engaging local supports across systems for families and children with complex needs.	
• Attitudes: Self Checking our responses to obstacles when family seeks help. What are we doing to make a system work for families who seek help?	
• How to avoid/manage misinformation.	
• Lots of navigational resources out there – making sure EVERYONE who needs to know, knows about them.	
• Can we develop Best Practice Guidelines for the practice of navigation?	
• How to create a Community or BC wide resource guide outlining community resources, grants, etc. for all special needs children/teens?	
• Breaking down silos: Can we create a visual map of BC family supports?	
• How do we engage with primary care providers to transfer knowledge about “navigators” to families?	
• How do we create a one stop shop for families?	

The Navigation Summit closed with words and reflections from Jason Gordon, Provincial Advocate with the BC Association for Child Development and Intervention. He thanked attendees for their participation and asked them to share their responses in Mentimeter to the question, “What are you most excited about for what ‘navigational supports’ for families, and a network of navigators, could be in the coming years?” Attendees’ posted comments centred on three areas. First, they expressed their hopes for improved and continued connection amongst service providers, including improved communication, understanding of each others’ roles, and opportunities for knowledge building and sharing (e.g., through a community of practice or other type of formal network of navigators, further meetings/summits, or working groups). They also hoped for improved connections specifically amongst rural families and professionals, an up-to-date and maintained provincial online mapping of resources, and better training to be able to provide navigation supports in culturally safe ways. Finally, they shared their hope that families would be empowered by being able to easily access consistent, connected, and seamless supports through a more simplified and streamlined system.

### Attendees’ evaluation of the Navigation Summit

Immediately following the conclusion of the Navigation Summit, attendees received an email containing a link to a short, online evaluation survey. Overall, approximately 50% (*n* = 64) of Summit attendees completed the survey, providing feedback on the various sessions they attended, as well as about the perceived impact to their practice with families. They were also asked to share general event feedback, such as the perceived utility of attending by Zoom, their experience in various breakout rooms, and about the amount of time spent each day. Overall, event feedback was very positive. Respondents indicated that they had gained an improved understanding of the challenges and complexities experienced by families trying to navigate service and support systems (85%), of the varied terminology associated with the term ‘navigation’ (75%), and of the mandates and scopes of service of different providers (75%). Attendees also indicated that what they had learned influenced how they intended to work with families going forward (63–73%). Over 80% of respondents also indicated that the ‘Creating Strategies Together’ session identified meaningful approaches to developing opportunities for interagency connection and collaboration, and was helpful for identifying priorities for the future. Feedback pertaining to event structure and delivery was also positive, as 90% indicated that Zoom was an effective way of facilitating the Summit, 95% indicated that they found the breakout rooms to be effective and meaningful, and 73% endorsed that the amount of time devoted to the Summit was ‘about right.’ Attendees also provided positive elaborative statements, such as:

*This was truly one of the best conference/summit I've been too. Technology actually was a benefit in this sense with the breakout rooms and using of the Mentimeter and Miro. Being able to pop out and around to other rooms at the end was in many ways, way more doable then if we were all in a room and getting up and moving. Loved the whole summit, connecting with so many different individuals and organizations. Was the best I've been to in a long time, and the whole event was so seamless and smooth.*

Another individual wrote:

*I found it so insightful and incredible to connect with others who are supporting families. It was also very well run even though it was virtual and both days were so engaging it actually felt like the time was flying by. Thank you for providing the opportunity to connect with so many people! It was motivating and I am eager to see what we can continue to do together to support families as well as make broader changes so that families are much better supported.*

### Navigation Summit next steps and summary infographic

**Fig. 3:**
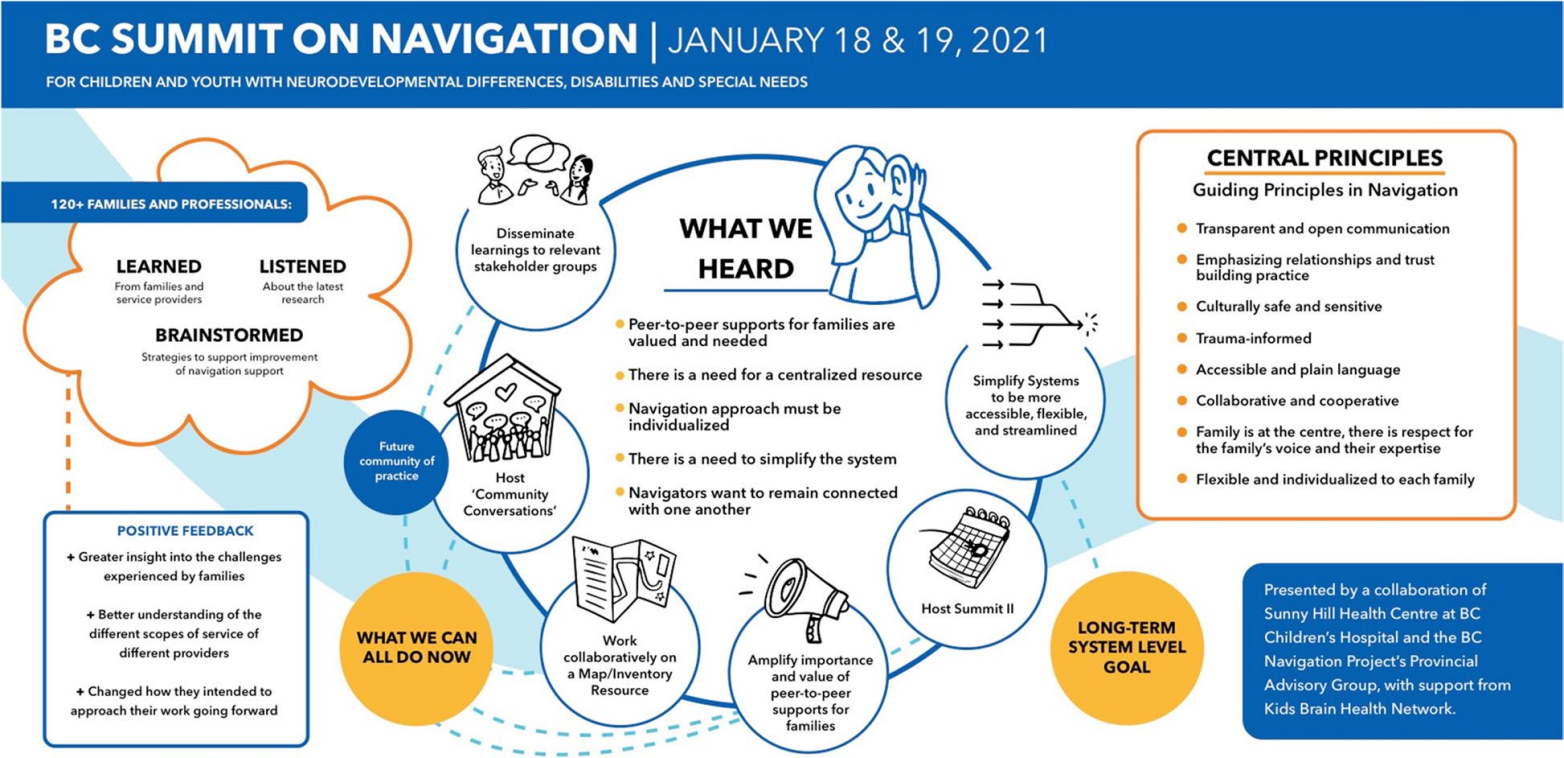
Summary Infographic of the Navigation Summit

Following the Navigation Summit, the planning team met to establish next steps, forming three working groups amongst team members. The first working group, ‘Making Sense of the Summit’, was tasked with analyzing the comments and posts (i.e., the ‘data’) obtained from the breakout rooms, Zoom chat, and various engagement opportunities through Miro and Mentimeter in order to inform the creation of a summary document that would showcase the main messages heard at the Navigation Summit. Within this working group, six team members independently analyzed all data, identifying main themes and messages from each discussed topic and breakout room. The group then came together to share their conclusions, and to reach consensus as to what the main messages were. Following this, the team reached out to a graphic designer, who created a summary infographic (see Fig. 3).

The second working group, ‘Building a Community from the Summit’, had a focus on creating ongoing linkages and communications with Navigation Summit attendees, on continuing to build a community of stakeholders across the province, as well as communication and data sharing with attendees. This group hosted two ‘Community Conversations’ events via Zoom, as a way of fostering continued connections amongst those who had engaged with the Navigation Summit. The first, which took place in June, 2021 explored the most popular resources navigators use when finding services for families, as well as how they appraise the integrity of available information. The second event in October, 2021 asked attendees to consider whether they would like to be involved in maintaining a network or community of navigators, and what would help to create and sustain such an endeavor. Each event was attended by 15–30 individuals.

The third working group, ‘Towards System Change’, was focused on strategic planning around how best to engage decision makers related to navigation and related supports for families of children with ND in BC. This group was successful in securing a meeting with Policy Managers within the Ministry of Children and Family Development (MCFD) in June 2021, within which learnings, recommendations, and future directions gleaned from the team’s work both broadly as part of the Integrated Navigational Support Program, as well as specifically from the Navigation Summit, were shared. Representatives from MCFD were keenly interested in the work presented; however, their ability to engage more directly with the findings and recommendations we identified was constrained by a coinciding policy effort revising the service delivery framework for children and youth with support needs.

## Follow-up activities

**Fig. 4:**
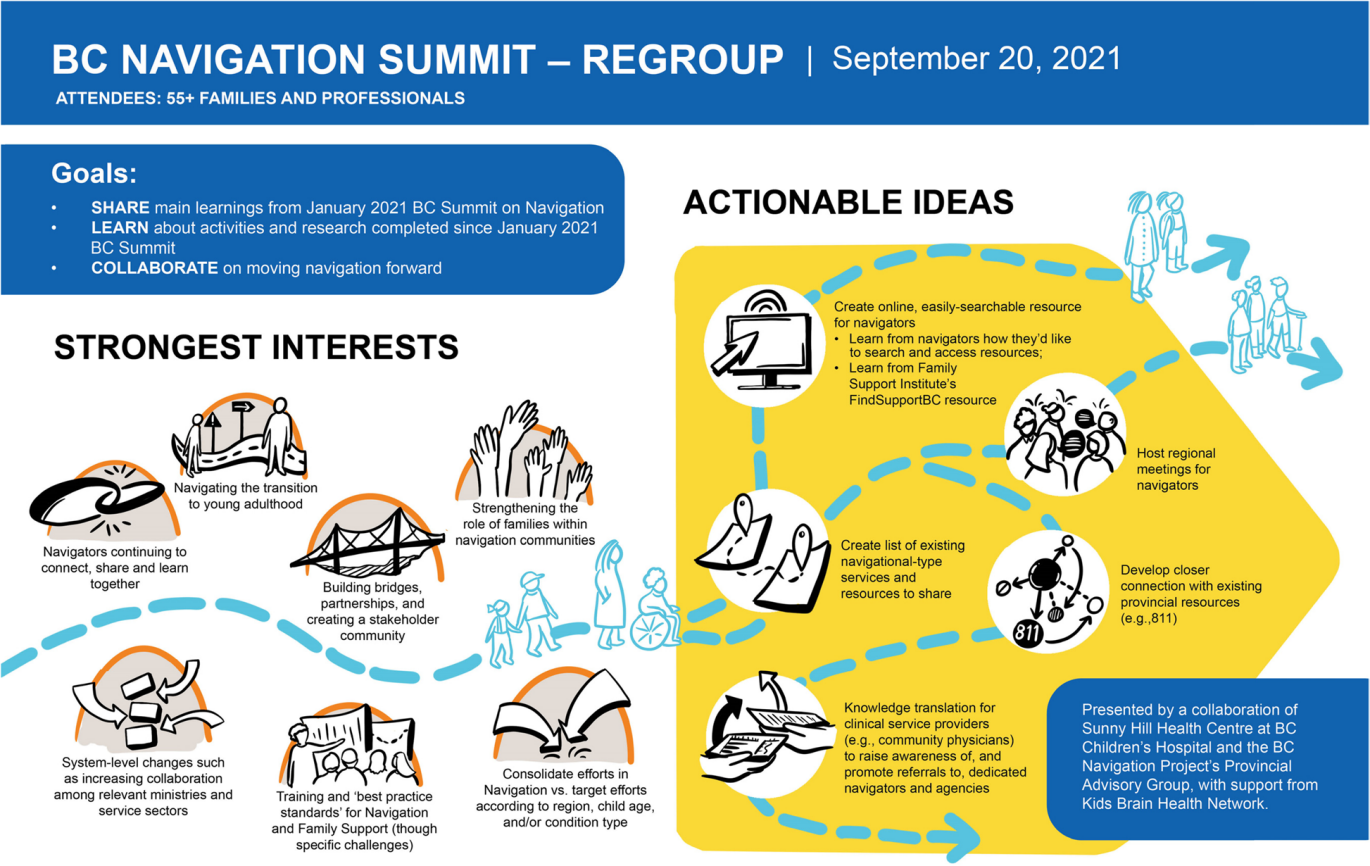
Summary Infographic of the Navigation Summit Regroup

Following the overwhelming message from Summit attendees that there was a desire to stay connected, and to have a venue to continue the conversations started, the team endeavoured to organize a follow-up event. On September 20, 2021, we hosted the *BC Navigation Summit Regroup* via Zoom. This follow-up event focused on reviewing together the main findings and shared knowledge that had emerged from the Navigation Summit in January. We also provided an update about continued activities and progress made. Much of the time together was dedicated to collaborating with attendees to explore how to keep a nascent navigation community together and thriving in British Columbia, and to gather any other new and compelling ideas for strengthening navigational supports on behalf of families with children living with ND. This event was attended by approximately 60 individuals, including family members and service providers. A visual summary of this event is represented in Fig. 4.

## Data Availability

Not applicable.

## Abbreviations

AIS BC: Autism Information Services British Columbia
BC: British Columbia
FSI: Family Support Institute of British Columbia
MCFD: Ministry of Children and Family Development
ND: Neurodisability
PAG: Provincial Advisory Group
PN: Patient Navigation
